# The Rise of a Legend: Lithium and the Extraordinary Story of Its Discovery

**DOI:** 10.3390/ph18081230

**Published:** 2025-08-20

**Authors:** Konstantinos N. Fountoulakis

**Affiliations:** 1 3rd Department of Psychiatry, School of Medicine, Faculty of Health Sciences, Aristotle University of Thessaloniki, 54124 Thessaloniki, Greece; fountoul@auth.gr or kostasfountoulakis@gmail.com; 2 Society for Neurosciences and Rehabilitation (E.N.A.), 54124 Thessaloniki, Greece

**Keywords:** lithium, history, bipolar disorder, discovery

## Abstract

Lithium is still the gold standard in the treatment of Bipolar disorder in adults. Despite the recent advances and the continuous increase in the use of antipsychotics and specific antiepileptics, it is still widely used despite not being promoted by the industry. Lithium was discovered in the early 19th century in a Swedish mine, and only a few decades later, it was discovered that it could be added to water so that urea could be dissolved with the formation of lithium urate. This was considered in the frame of the uric acid diathesis theory, which, during the 19th century, was considered a strong etiopathogenetic theory for many pathological conditions, including mental disorders. The first attempts to use lithium as a treatment modality in psychiatry appeared in the late 19th century by Hammond and the Lange brothers, and during the late 19th century and the early 20th century, lithium was used in various remedies and beverages until its toxicity became evident. Lithium was established as a valid treatment option for Bipolar disorder only during the second half of the 20th century after the works of John Cade, Mogens Schou, Poul Baastrup, and others who pursued a line of research, frequently against the scientific opinion of the majority of colleagues.

## 1. Introduction

Lithium is still the gold standard in the treatment of Bipolar disorder. Despite recent advances in the field and the increased use of antipsychotics and specific antiepileptics, it is still widely used despite the lack of promotion by the industry. Its history is of particular interest, and reviewing it could lead to insights into how our understanding of mental illness evolved and the modern era of psychopharmacology emerged. Lithium carbonate is now on the World Health Organization’s list of essential medicines.

The current paper aims to give technical and historical information on this extraordinary and mysterious medication.

## 2. What Is ‘Lithium’?

**Figure 1:**
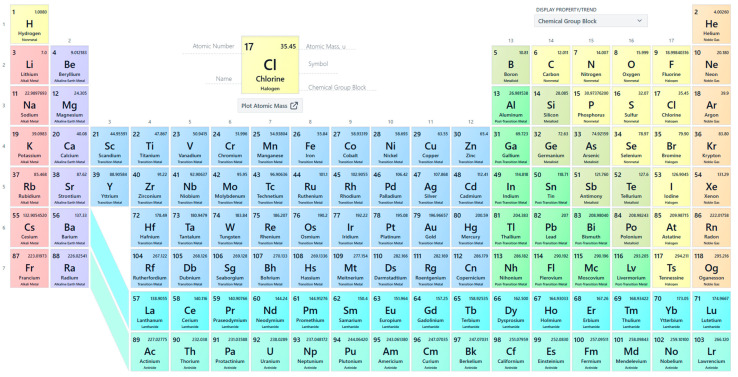
The periodic table, from https://pubchem.ncbi.nlm.nih.gov/periodic-table/ (accessed on 15 August 2025).

Lithium is a relatively rare chemical element (16 ppm in the Earth’s crust, 0.006 ppm in the universe) [1]; its symbol is ‘Li’. It belongs to the alkali metal group (Figure 1); it is the least dense solid element and the lightest metal in the periodic table [2]. Lithium is silver–white and soft enough to cut with a knife. Its standard atomic number is equal to 3, and its atomic weight is equal to 6.941 g·mol^−1^. Its melting point equals 180.5 °C (356.90 °F), and its boiling point is 1342 °C (2448 °F), and both are among the lowest points for a metal. Due to its low atomic mass, lithium has high electrode potential because it has a high charge and power-to-weight ratio. Lithium has good heat and electric conductivity in its metallic form, enabling it to store and transmit energy. This makes it well-suited for the building of rechargeable batteries. There are two stable lithium isotopes in nature (Li_6_ and Li_7_, with the latter accounting for 95.15%).

**Figure 2:**
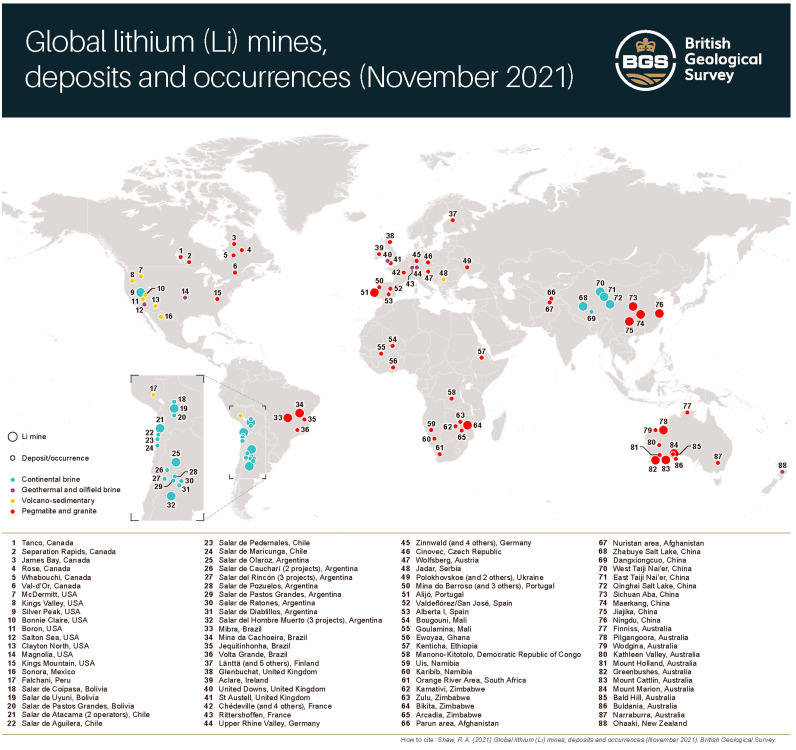
Global lithium mines and deposits according to the British Geological Society. From Shaw, R.A. (2021) Global lithium (Li) mines, deposits and occurrences (November 2021). British Geological Survey.

Lithium metal is highly reactive. It ignites in contact with water. As a result, in nature, it is only found as a mineral or salt and almost never in a pure form. Metallic lithium is soluble in short-chain aliphatic amines, e.g., etilamine. On the contrary, it is insoluble in hydrocarbons. Today, minerals rich in lithium, including spodumene and petalite, are mainly extracted from pegmatites in Australia, Zimbabwe, and Brazil (Figure 2) [3]. Other sources of lithium include oilfield brines, clays, and geothermal brines.

## 3. The History of Lithium

### 3.1. The Discovery (First Half of the 19th Century)

**Figure 3:**
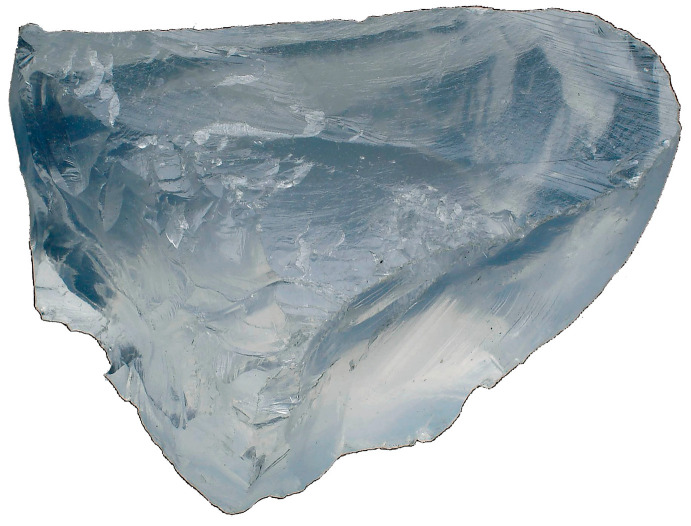
Petalite, the mineral where originally lithium was discovered, from Minas Gerais State, Brazil. Photo by Eurico Zimbres. Copyright status: CC BY 2.0.

Lithium was discovered by José Bonifácio de Andrada e Silva (1763–1838), a Brazilian chemist and statesman, in 1800, in a mine on the island of Utö, Sweden, in the form of petalite (LiAlSi_4_O_10_; Figure 3) [4].

It was re-discovered in 1817, this time by Eric Thomas Svedenstjerna (1765–1825), a Swedish metallurgist. Svedenstjerna sent samples to the laboratory of Jöns Jacob Berzelius (1779–1848), the foremost Swedish chemist of the day. There, Johan August Arfwedson (1792–1841) analyzed it, and he found that 96% of the petalite consisted of alumina and silica. The remaining 4% was an alkaline substance that did not respond to the usual tests for known alkaline elements. Eventually, Arfwedson identified a new element, and Berzelius, being the head of the laboratory, gave it the name ‘lithion’ or ‘lithina’, from the Greek word ‘λιθoς’ (‘lithos’ meaning “stone”). The name reflected its discovery in a solid mineral. On the contrary, potassium was discovered in plant ashes, while sodium is highly abundant in animal blood [5,6].

Both Arfwedson and Gmelin tried but failed to isolate the pure lithium from its salts. The pure element was isolated in 1821 by William Thomas Brande (1788–1866) by electrolysis of lithium oxide. In 1855, Robert Bunsen (1811–1899) and Augustus Matthiessen (1831–1870) produced larger quantities of lithium through the electrolysis of lithium chloride [7]. After the discovery of this procedure, the German company Metallgesellschaft AG started the commercial production of lithium in 1923 [8].

### 3.2. The ‘Uric Acid Diathesis’ Theory (Second Half of the 19th Century)

Already in the mid-nineteenth century, several medical practitioners noted that lithium compounds could dissolve uric acid. Lithium combines with uric acid to form lithium urate (C_5_H_3_LiN_4_O_3_), a soluble salt. At the time, prominent doctors believed that excess uric acid was the culprit behind many severe illnesses. So, based on its ability to dissolve uric acid, physicians of the mid-19th century began treating many diseases, including asthma, diabetes, obesity, indigestion, rheumatism, skin disorders, and headaches, with lithium.

In 1841, Alexander Lipowitz (1811–1873), a German chemist, pulverized some lepidolite, an ore containing lithium, mixed it in boiling water with barely soluble uric acid, and found that he had produced the far more soluble lithium urate. Two years later, Alexander Ure (1808–1866), a British surgeon and father of the field of drug metabolism [9], during a lecture to the Pharmaceutical Society, showed how lithium carbonate, when put together with uric acid crystals, formed lithium urate. This mechanism dissolves uric acid crystals. Ure had a particular interest in finding a remedy for gout and for the urinary stones that often accompany it, at least partially because his father, an eminent scientist as well, had suffered from this disease for many years. His observation suggested that injecting lithium carbonate directly into the bladder might be a good way of reducing the size of bladder stones [10]. It took 16 years, but finally, in 1859, Ure was able to get his hands on enough lithium carbonate to try it as a treatment on a 56-year-old male patient with a large and uncomfortable bladder stone. He injected a solution of lithium carbonate directly into the bladder. He did this every other day for several weeks. Disappointingly, the stone did not get any smaller. To ‘expedite matters’, Ure resorted to crushing the stone with a lithotrite, an instrument inserted into the bladder. After performing this several times, he had broken up much of the stone and a successful conclusion seemed likely. Unfortunately, before Ure removed the final fragments of stone, the patient died. In his report of the case, Ure wrote that at least ‘the solvent had in some measure lessened the cohesion of the concretion and increased its friability’ [11].

Sir Alfred Baring Garrod (1819–1907) was a prominent British physician who announced that he had discovered uric acid in the blood of gouty patients. He was a contemporary of Ure and was familiar with Ure’s bladder stone work. He repeated his original experiment with a gouty bone instead of a bladder stone. He placed a finger bone, which featured a prominent gouty (uric acid) deposit at the joint, in a solution of lithium carbonate. Within several days, the deposit had disappeared. Garrod propagated the notion that excess uric acid was behind an assortment of other medical conditions, from headaches to diabetes, also including many mental disturbances.

It is interesting that according to the works of Armand Trousseau (1801–1867) in France and Alexander Haig (1853–1924) in the UK, mania and depression were also thought to be related to uric acid (brain gout). Being a prominent Scottish physician, Alexander Haig is said to have cured his migraines by switching to a vegetarian diet, which is low in uric acid. Subsequently, based on his personal experience, Haig concluded that behind all illnesses, there was a uric acid disorder that served as the cause. He described it as ‘uric acid diathesis’ (a tendency to produce too much uric acid) [12,13]. This concept, the ‘uric acid diathesis’ theory, captivated the medical profession. Garrod advised that lithium carbonate be taken orally as a treatment for gout as well as for other uric acid diathesis conditions. By the end of the 19th century, influential physicians throughout Europe and the US had embraced the concept and strongly recommended lithium for a variety of conditions. Unfortunately, eventually, it was proven that in order to dissolve urate in the body, the lithium levels needed would be toxic [14,15,16,17]. The uric acid diathesis remained a leading theory about the cause of disease well into the 20th century.

Garrod and other advocates of the uric acid diathesis believed that mental symptoms commonly went along with gout and excess uric acid. They thought that uric acid attacked the brain and, as a result, caused all sorts of mental disorders, including melancholy and, as Garrod put it, ‘gouty mania’. Garrod and others treated all signs of uric acid diathesis with lithium compounds and reported that, along with other symptoms, mental ones improved as well.

The first neuropsychiatric indication for lithium came from the eminent American neurologist Silas Weir Mitchell (1829–1914). In 1870, Mitchell recommended lithium bromide as an anticonvulsant and hypnotic [18] and later for ‘general nervousness’ [19]. It worked fairly well, probably because bromine is a good sedative, and it continued as an epilepsy treatment well into the 20th century. Mitchell also gave lithium bromide to people with an assortment of nervous symptoms, some of whom were probably suffering from depression, and reported that they received relief. Again, the relief may have come from the bromine portion of the compound, not the lithium.

**Figure 4:**
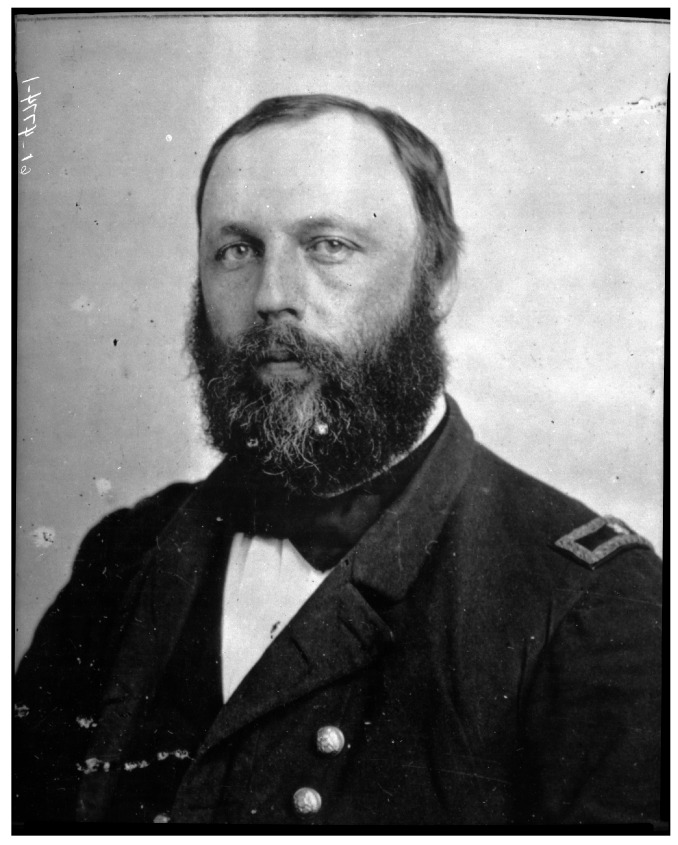
William Alexander Hammond (1828–1900).

Then, in 1871, William Alexander Hammond (1828–1900; Figure 4) was maybe the first to prescribe a modern and effective psychotropic agent specifically for a mental disorder, and this was lithium for acute mania [20]. At that time, he was a professor of Diseases of the Mind and Nervous System at the Bellevue Hospital Medical College in New York. In his 1871 textbook on diseases of the nervous system, Hammond reported that he had used lithium bromide to treat patients with acute mania and discovered that it rapidly and effectively calmed them. He used exceptionally high doses. It is not clear whether the lithium or the bromine part produced the results. Additionally, it was unclear if some kind of sedation due to toxicity accounted for this. In his later textbooks, published in 1882, 1883, and 1890, Hammond did not mention the use of lithium, suggestive of a disappointment [21,22]

**Figure 5:**
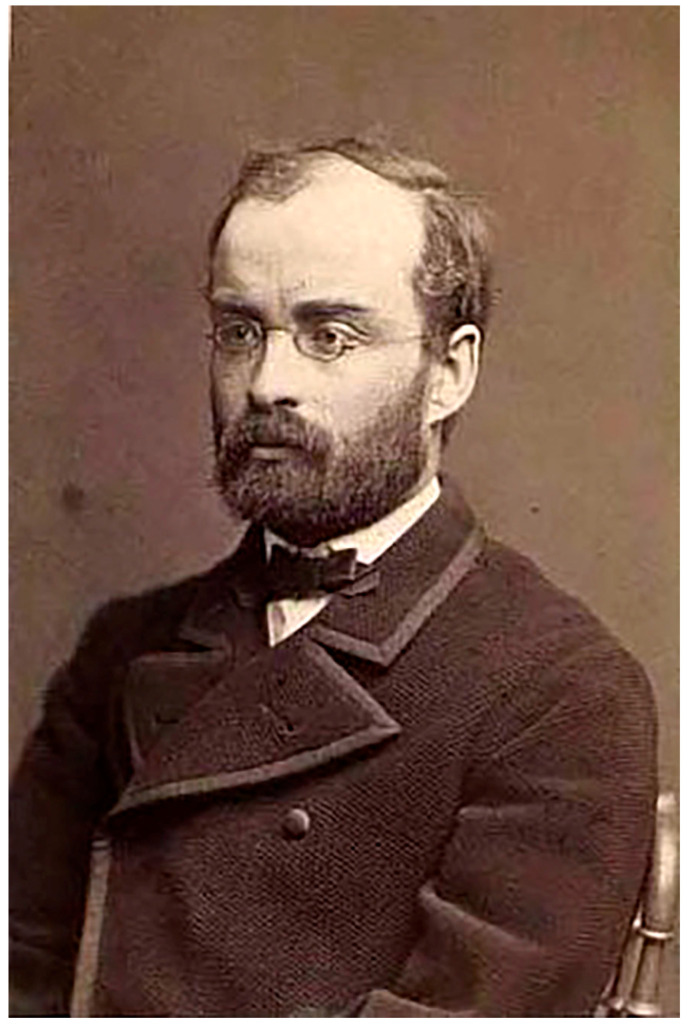
Carl Georg Lange (1834–1900).

A few years after Hammond, the Danish psychiatrist Carl Lange (1834–1900; Figure 5) used lithium in the treatment of recurrent brief depression in 1886, while his brother Frederik Lange (1842–1907) used lithium in the treatment of 35 patients with melancholic depression (including some milder forms of Bipolar disorder) in 1894 [23]. In 1886, the year after he published his work on emotion, he came out with a treatise on what he called periodical depressions. In it, he described a series of patients he had cared for in his medical practice who had repeated depressions of milder severity than is usually seen by psychiatrists. He noticed what he thought was an unusual sediment in these patients’ urine and assumed, incorrectly, that the sediment was uric acid. Lange went on to hypothesize that these depressions were caused by excess uric acid and suggested that they could be treated and prevented by the conventional (at the time) therapies for uric acid conditions, which included lithium carbonate. In later writings, he explicitly mentioned lithium carbonate as a treatment for these ‘uric acid depressions’. The lithium doses he advised were comparable to those used today for Bipolar disorder, and Lange reported favorable outcomes with this regimen. Lange’s colleagues in Denmark knew of his work, but for the most part, the larger international psychiatric community did not.

Eventually, despite encouraging results, by the turn of the 20th century, the ‘brain gout’ theory of mood disorders disappeared as a medical concept, and the use of lithium in psychiatry was abandoned.

### 3.3. Remedies and Beverages (Late 19th Century and First Half of 20th Century)

However, while the scientific medical community rejected the gout theory, Haig’s writings were extremely popular with a large percentage of the lay public. With the advent of uric acid diathesis as the explanation for an assortment of illnesses and the widespread application of lithium as its treatment, the promotion of mineral waters now had a ‘scientific rationale’. By the last decade of the 19th century, people were flocking to mineral springs and the resorts that sprang up around them. Some of these springs contained trace amounts of lithium and some no lithium at all, but these ‘lithium waters’ were sought as a cure for rheumatism, diabetes, asthma, gout, and every other imaginable ailment. People also prized them for what were widely believed to be their general health-promoting properties, and both bathed in and drank the waters. ‘Lithia water’ became widely available to the general public without recommendation or prescription. Innumerable brands of bottled lithium water came on the market, each promising extraordinary health benefits. Multiple companies sold water labeled as containing lithium, one of which continues to operate as a business presently.

**Figure 6:**
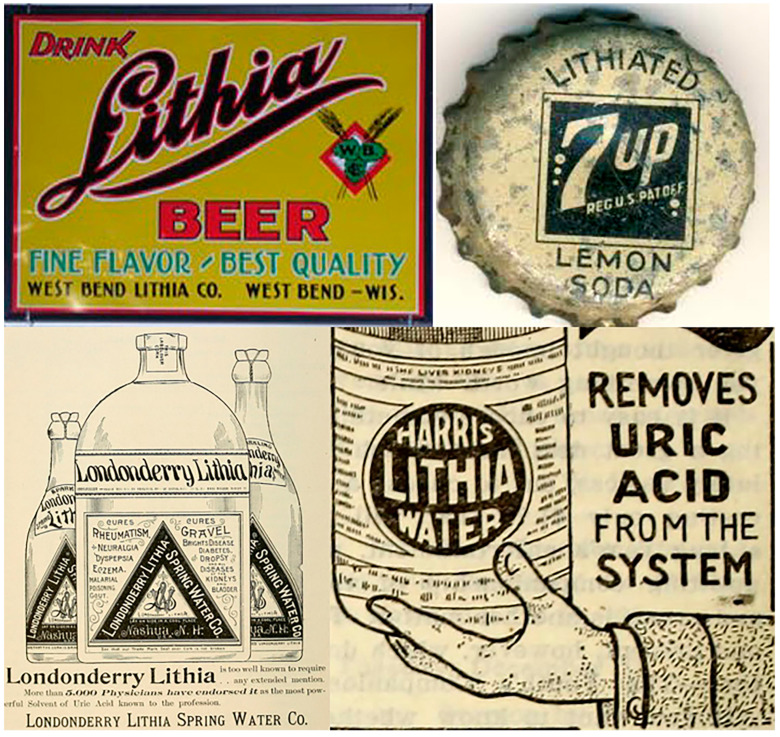
Examples of beverages which included lithium as a component in the late 19th and early 20th centuries. Left upper corner: Lithium beer. Right upper corner: The original 7UP which included lithium. Bottom: An example of remedies with lithium which were marketed in the late 19th and early 20th centuries and were mostly indicated for the control of renal calculi and ‘uric acid diathesis’.

Also, several beverages included lithium as a component in the late 19th and early 20th centuries (Figure 6). Charles Leiper Grigg (1868–1940) introduced a lemon–lime soft drink in 1929 under the label ‘Bib-Label Lithiated Lemon-Lime Soda’, which soon changed to 7UP. Lithium citrate was one of its seven main ingredients and a key selling point. Soon after launch, its inventor changed the name to 7Up Lithiated Lemon Soda, and in 1936, he shortened the name to 7 Up. The lithium was not removed until 1950. Also, Coca-Cola offered a lithium-containing version that did not last long. Eaux de Vichy is another example of natural water advertised as containing lithium.

Later studies eventually showed that lithium taken by mouth has no effect on uric acid or its elimination, partly because other compounds circulating in the body interfere with lithium’s dissolving ability. By 1948, lithium had been removed from all beverages because of cases of toxicity, and its free marketing was prohibited [14].

### 3.4. The Second Half of the 20th Century and the Re-Discovery of Lithium

**Figure 7:**
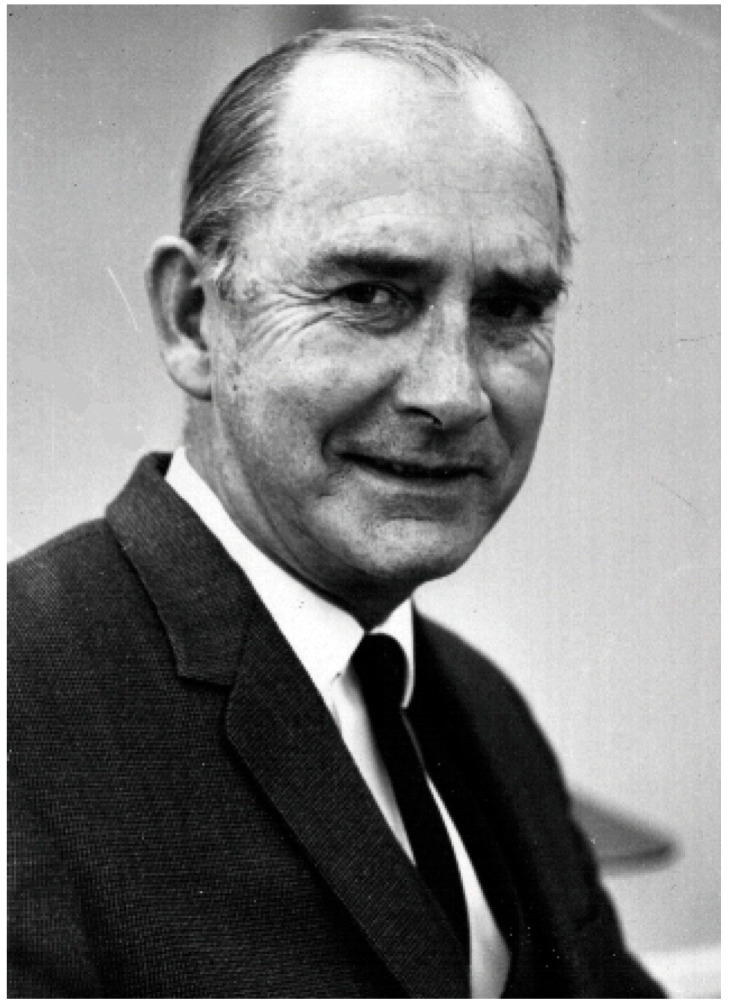
John Cade (1912–1980) from the Herald & Weekly Times Portrait Collection, State Library of Victoria, Australia.

After World War II (WWII), a boom in biomedical research appeared that eventually led to the emergence of the modern era of psychopharmacology. Although this era of psychopharmacology is considered to have begun in the 1950s with the introduction of antipsychotics, in reality, it started a decade earlier with the re-discovery of lithium by John Cade (1912–1980; Figure 7) in 1949 [15].

John Cade was born on 18 August 1912, in a country town called Murtoa near Melbourne in Australia. Cade’s father, David Cade, worked there as a General Practicioner, and his mother worked there as a nurse. Not psychologically able to deal with the demands of his position due to Post-Traumatic Stress Disorder (PTSD) from World War I (WWI) and the Spanish flu pandemic, David Cade sold his private practice and started working as an asylum doctor in Victoria’s Mental Hygiene Service. For the next decades, he served as the medical superintendent at several Victorian mental hospitals. An interesting detail is that at that time, it was customary for a house within the grounds of the asylum to be provided to the superintendent and his family. This led John Cade to develop a deep understanding of the needs and the burden of mental patients and their families [24].

By 1936, at the age of 24, Cade was appointed to Beechworth Mental Hospital, the same hospital where his father had worked and the Cade family had lived 15 years earlier. Three years later, he moved to the department’s Bundoora Repatriation Mental Hospital. There, he had an exceptional experience in research since, during a flu outbreak, he worked with Sir Frank Macfarlane Burnet (1899–1985), an eminent virologist and immunologist. Burnet received the Nobel prize in 1960. The research project concerned measuring antibodies in the blood of afflicted patients [25].

With the break of WWII, Cade joined the army, and in September 1941, he was promoted to major. Then things went badly as Singapore fell to the Japanese on February 15th, 1942, and Cade was taken as a prisoner of war along with around 80,000 RANZAC soldiers. Cade was put in charge of the psychiatric section in a hospital organized in the Changi prisoner of war camp, and there he began to conceptualize a relationship between diseases and food deficiencies in a way parallel to that of Archie Cochrane in the frontstalage 183 German prisoners of war camp in Thessaloniki, Greece. During that period, he also developed the solid idea that severe mental disorders have a physical basis.

**Figure 8:**
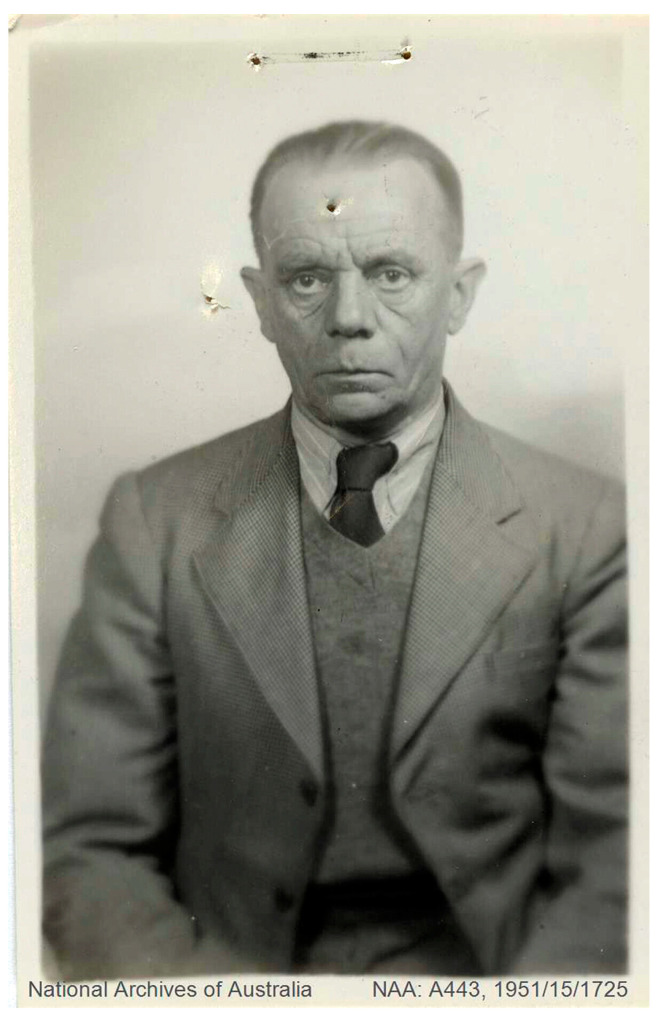
Eduard (later known as Edward) Trautner (1890–1978).

A second critical figure in the re-discovery of lithium was Eduard (later known as Edward) Trautner (1890–1978; Figure 8) [26]. Trautner was of German origin and was a scientific polymath; he studied several sciences, including animal physiology, botany, and biochemistry. He arrived in Australia on the HMT Dunera, a ship that achieved notoriety for the appalling conditions of its voyage to Sydney, in early September 1940 with another 2542 so-called enemy aliens who were deported to Australia by the British government. Most of them were Jewish refugees, and others who opposed the Nazi regime and had fled Germany. After arriving in Sydney, he was sent to an internment camp in Tatura, central Victoria, where he was discovered by the eminent professor of physiology at the University of Melbourne, Sir (AK) Roy Douglas ‘Pansy’ Wright (1907–1990). Wright arranged for Trautner to join Melbourne’s physiology department in 1942. Trautner worked there for the next few years, developing procedures aimed at extracting pharmaceutical agents from plants.

After the war, John Cade started working on research using abandoned spaces in Bundoora Repatriation Mental Hospital near Melbourne. Cade had little formal research training, and he went on to create a makeshift laboratory on the grounds of a small, isolated mental asylum in an unused kitchen. He had no access to sophisticated chemical analysis and no theory to guide him. He had neither funding nor collaborators.

His belief that there is a physical dysfunction underneath mental disorders led him to the assumption that severe mental disorders occur because the body produces a high amount of an otherwise normal substance, and he formulated a theory in analogy with hyperthyroidism, for whom medication treatment had started emerging around that time. According to this conceptualization, schizophrenia and mania were the result of the excess of this substance, while melancholia was its deprecative condition.

Since he had no idea what the substance might be, he thought that the best way to identify it would be by studying conditions like mania and psychosis, where this substance is supposed to be an excess. In this line of thought, if such a substance does exist in excess, then either itself or one of its metabolites would be present in the urine in excess. With this in mind, Cade started collecting early-morning urine samples from patients as well as from healthy persons because these urine samples were quite concentrated since they were passed after 12–14 h without fluid intake. Theoretically, the suspected toxic substance would exist in its highest concentration in these samples. The next step was again a step into the unknown. The substance was unknown, and its physiological actions were also unknown. If it was toxic when in excess, then some kind of toxicity test could reveal it. Cade decided to go on with a very crude toxicity test, and in 1949, he simply injected various amounts of urine into the abdominal cavities of guinea pigs. He titrated the dosage and determined the quantity required to kill them. He observed (mistakenly) that the urine of people with mania was especially lethal to the animals. He registered that the urine from manic patients would kill guinea pigs in a dose as low as 0.25 mL per 30 g of body weight, whereas the lethal dose of the most toxic urine from patients with other diagnoses and healthy people was at least 0.75 mL per 30 g of body weight. However, the mode of death was the same, regardless of the source of the urine, pointing to the possible conclusion that there was an issue of quantity, not quality. Twenty minutes after injection, the animal started to tremble and lose its balance; convulsions followed, leading to coma and death in a state of status epilepticus. To identify the toxic substance, Cade embarked on a series of experiments without following an entirely logical or readily understood sequence; most likely, they were a kind of fishing expedition. Even ‘worse’, in the process, Cade misinterpreted critical aspects of the observations.

His first step was to test the toxicity of the common contents of urine, which are urea, uric acid, and creatinine, which are all normal products of protein metabolism. After injecting high concentrations of each of them into the abdominal cavities of guinea pigs, he observed that creatinine and uric acid were harmless, but on the contrary, urea killed the animals in the same manner urine did. This could mean that urea was the substance responsible for the lethal effect of urine. The problem was that there was no higher concentration of urea in the urine of manic patients in comparison to the other diagnostic groups, and additionally, that concentration was not high enough to produce this lethal effect. Taken together, all these factors meant that there was probably another unknown substance in urine that was responsible for the deaths of the animals. That unknown substance could either act independently or enhance the lethality effect of urea. His next step was to try combinations of the components in urine, but this led nowhere. In fact, he discovered that creatinine prevented convulsions and death caused by urea, and after further pursuing this pathway, Cade was able to show some anticonvulsant efficacy of creatinine [27]. It is not known whether this is valid since nobody ever tried to replicate these results. Another combination would be urea plus uric acid, but here, there was a practical problem: uric acid does not readily dissolve in water. One of the best solutions to this problem was to use the most soluble salt of uric acid, lithium urate. Again, surprisingly, lithium urate reduced urea toxicity, and when Cade decided to clarify whether this was due to lithium rather than to uric acid, he discovered that this was the case with other lithium salts like lithium carbonate, which also seemed to have protected guinea pigs from urea’s toxicity. Further testing confirmed the protective effect of lithium salts. Cade also noted a suppressive and calming effect of lithium on animals and described the animals after lithium injection as ‘extremely lethargic’, but returning to normal after one or two hours. In fact, it is possible that the animals were lethargic because of lithium toxicity. All the facts suggest that these guinea pigs endured not a beneficial tranquilizing effect of lithium but rather early signs of lithium poisoning, which typically include lethargy and lack of responsiveness. Other investigators who later gave guinea pigs and other animals lithium were unable to reproduce the inactivity that Cade observed unless they injected the animals with large toxic doses.

The discovery of lithium as a potential therapeutic agent led naturally to the next step, which was to use it on patients. It is not known whether this decision was also supported by knowledge of the previous history of lithium as a treatment option during the late 19th century. It is a fact, however, that Cade ‘arbitrarily’ selected the doses of lithium that he initially gave to manic patients based on the doses that had been used during the previous century to patients suffering from diseases presumably caused by uric acid. This is similar to the pathway followed by Hammond since the dosages reported by both him and Cade lead to lithium intoxication. Interestingly, Cade himself suggests that he just got lucky. In reminiscing about his discovery 20 years later, he said, ‘The original therapeutic dose decided on fortuitously proved to be the optimum, that is 1200 mg of the citrate thrice daily or 600 mg of the carbonate’. But given the effectiveness of the dose he decided on, some have speculated that along with Cade’s good luck, a bit of conscious deliberation may have been at play.

Cade took lithium himself to see if it would cause adverse effects and to establish the correct dosage before administering it to patients. He took single and repeated doses of lithium citrate and lithium carbonate starting in early February 1948 and continued to do so for two weeks. Cade did not record what doses he consumed but recounted that they were the doses he thought would be appropriate to give to patients. First, he tried lithium on himself to establish how high a safe dose could be. Then, Cade began treating ten people with acute mania.

His first patient, known only by his initials, WB, and his first name (William or Bill), had suffered symptoms of mania and depression for 30 years and had been confined to Bundoora Hospital for the past five years with unrelenting symptoms of mania. After his excellent response to lithium and discharge from the hospital, he was readmitted six months later with severe mania. His brother reported that he had become overconfident about having been well for so many months, became reluctant to take medication, and eventually ceased taking lithium about six weeks before. Subsequently, he had become increasingly more irritable and erratic. Once lithium was readministered, within two weeks, he had again returned to normal, and he was released again a month later.

Over the following year, Cade went on to treat nine more manic patients with lithium and saw equally good results. In September 1949, he published a paper reporting both fast and dramatic improvements in all of them [28]. The most important observation was that while the majority of them had been in and out of Bundoora for years after the treatment with lithium, five of them had improved enough to return to their homes and families. Cade was 37 years old when his 1949 paper appeared. He never did any further research on lithium. The last survivor of the original 10 manic patients died of natural causes in 1980 at the age of 76. He had been on lithium for more than 30 years. John Cade died the same year.

Sadly, two years after the publication on the successful use of lithium, the first death because of lithium toxicity was reported in a Bipolar patient who otherwise responded extremely well to treatment. This first death was Cade’s first patient, WB, who had a rocky time over the two years after the publication. He remained outside the hospital for months at a time and worked intermittently, but he did not take lithium regularly and vacillated between periods of mania when he was off lithium and stomach upset when he was on it. In March 1950, noting that WB still had ‘dyspepsia, anorexia, and vomiting’, Cade stopped his lithium. ‘Under all circumstances’, Cade wrote, ‘it seems that he would be better off as a free restless case of mania rather than the dyspeptic, frail little man he looks on adequate lithium’. Then, a couple of weeks later, he noted, ‘he was rapidly reverting to his manic phase and forgetting his dyspepsia’. One month later, in the face of WB’s unrelenting mania, Cade seemed to change his mind. In his note of 3 May 1950, he wrote that WB ‘has continued manic, restless, euphoric, noisy, dirty, mischievous, destructive, flight of ideas and thoroughly pleased with himself. His state seems as much a menace to life as any possible toxic effects of lithium. Therefore, recommence lithium citrate gr. 20 tds. Today’. Within two weeks, WB was ‘quieter but miserable and asthenic’. Cade discontinued the lithium, but WB started to go downhill. Over the next few days, he became semicomatose and had three seizures. WB died on 23 May 1950. Cade recorded as the primary cause of death ‘toxaemia due to lithium salts, therapeutically administered’. It is to be noted that he never properly reported this death and the probable cause. WB’s name exists in the coroner’s records of that year, and the cause of death recorded is lithium poisoning [29,30,31]. Cade’s worry about toxicity prompted him to stop studying and using lithium soon after he completed his clinical trial and to discourage others from using it as well, so in 1950, he abandoned his experiments with lithium. Cade’s 1949 paper went largely unnoticed at the time. Searching for an element with similar therapeutic properties but less toxic, Cade experimented with salts of rubidium, cerium, and strontium. None proved therapeutic. 

Interestingly, WB is the only patient that Cade portrays in his accounts; as a result, WB has achieved a sort of prominence as a poster child for lithium, or at least Cade’s poster child, despite his death. Schioldann wrote that Cade’s presentation of the case of WB was eventually misleading since he failed to explicitly report his death because of lithium toxicity [32]. We will never know if Cade’s silence on this matter was a deliberate attempt to conceal a painful truth or, in part or whole, an unintentional lapse.

While Cade was publishing his findings, Edward Trautner at the Department of Physiology and Pharmacology at the University of Melbourne, realized that he could measure blood levels of lithium using flame photometry. Charles Noack (1917–1969), a new psychiatrist on the staff of Melbourne’s Mont Park Hospital and Trautner’s former student, had heard of Cade’s lithium work and was interested in examining what seemed like a promising treatment. Noack turned to Trautner for help in planning and implementing a lithium investigation. Trautner quickly understood the importance of the matter and that the management of toxicity would be the key. Together with Noack, they proceeded to carry out a seminal study, which is, however, not as much historically appreciated as it deserves, although it is one of the founding stones of lithium treatment. They started with the same lithium doses used by Cade and carefully monitored patients for signs of lithium toxicity. They reduced the dose or temporarily stopped the lithium entirely if symptoms of toxicity occurred. Mindful of Talbott’s findings [33] on the relationship between lithium poisoning and serum levels, they decided, along with meticulous observation of patients’ symptoms, to measure lithium serum levels. This was the first attempt to measure serum lithium levels during lithium treatment of mania. It was made possible thanks to Victor Wynn (1920–2006), one of the pioneers of the study of metabolism [34]. At that time, Wynn was a young Research Fellow in the Physiology Department. At that time, the available techniques to measure electrolytes were cumbersome, inaccurate, and time-consuming, so Wynn personally raised the money to buy a flame spectrophotometer, a new device recently introduced by the Beckman Instrument Company, which made it possible to measure electrolytes speedily and with high precision. This flame spectrophotometer, which Wynn and his group were using to quantify sodium and potassium in biological fluids, could also be used to measure lithium, a similar chemical. Trautner and Noac were thus able to measure lithium levels and carefully monitor and adjust the lithium dose [35]. Their study established several key points, all of which have withstood the test of time. First, lithium, just as Cade had said, alleviated symptoms of mania. None of the 100 patients in their study (more than 30 of whom had acute mania) suffered serious intoxication, and none died. All but one of their more than thirty manic patients improved, and many continued on lithium and remained well during more than a year of observation. Noack and Trautner also recorded lithium’s preventive efficacy against mania, and in accord with the observation by Cade, they confirmed that it had little impact on depression or the fundamental symptoms of schizophrenia. Finally, Noack and Trautner showed that serum lithium levels could be practically monitored. The levels they observed were consistent with Talbott’s; the average was about 1.0 mEq per liter, and the highest level they saw was 2.1 mEq per liter in a patient with no signs of poisoning. In this and subsequent studies, Trautner and his colleagues identified a specific lithium blood-level range necessary for both safety and effectiveness [36,37,38]. Although Trautner’s work made it possible for lithium to be widely studied and prescribed, his contributions have barely been acknowledged.

**Figure 9:**
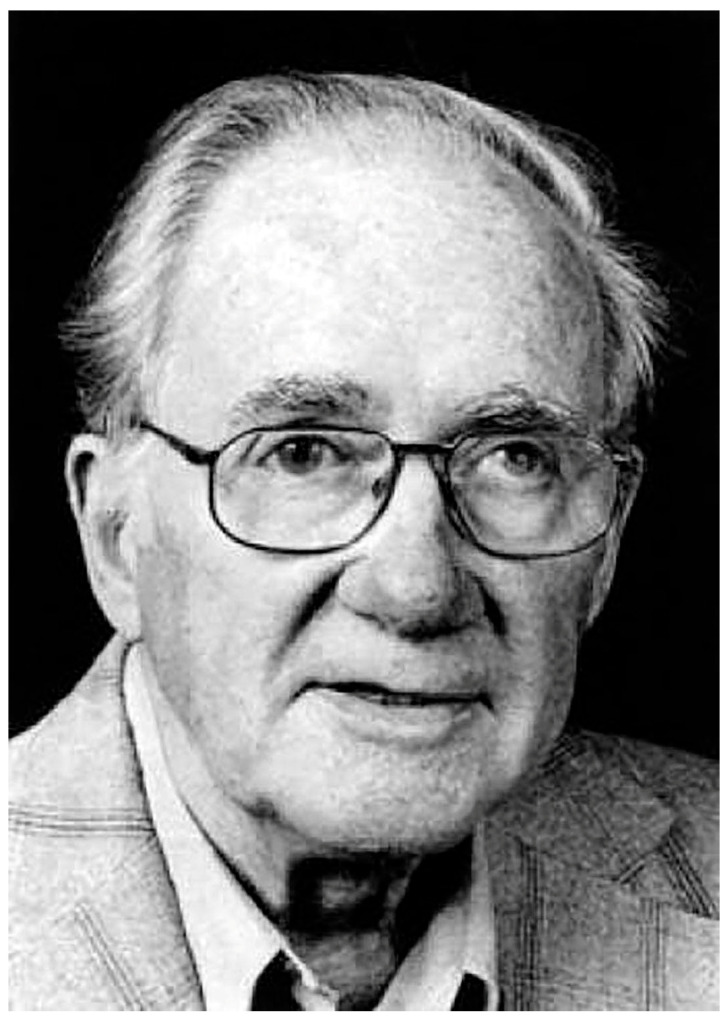
Mogens Schou (1918–2005) with permission from the INHN.

While Cade decided to abandon lithium as a treatment option, Noack and Trautner’s paper received the attention of the Danish physician Mogens Schou (1918–2005; Figure 9). Mogens Schou was not a psychiatrist but a physician who specialized in clinical chemistry and had observed a dramatic therapeutic effect of long-term lithium treatment in his younger brother. Erik Stromgren (1909–1993), head at that time of the Aarhus University psychiatric clinic in Risskov, asked him to undertake a randomized controlled trial of lithium in mania [39,40,41].

Schou proceeded to conduct the first controlled study of lithium in acute mania. He randomized acutely manic patients to lithium or placebo with a flip of a coin, and every two weeks, placebo and lithium alternated. Some of the patients entered the study using proper randomized controlled trial (RCT) rules. In 1954, he published the results, which made a significant impact [42]. The RCT is now the gold standard for treatment research; since the late twentieth century, it has been the most widely used method, and it is absolutely required to assess and confirm the value of new treatments. But in the early 1950s, when Schou did his lithium study, the RCT was a fairly new approach that had only recently been applied in medical research (the first published RCT, a study of streptomycin in tuberculosis, came out in 1948). Before this time, new treatments were assessed, if they were assessed at all, more or less the way Cade had evaluated lithium. Schou and his colleagues confirmed the value of lithium in treating mania by carrying out one of the first RCTs, if not the very first RCT, in psychiatry. The result, which verified lithium’s unique healing properties, was a turning point for lithium and psychiatry [42]. Schou measured serum lithium levels, and he found that they usually ranged from 0.5 to 2.0 mEq per liter and were consistent with Noack and Trautner’s observations in their first study. Again, in agreement with them, Schou did not observe a strong correlation between serum lithium and signs of toxicity [42]. Of utmost importance was his observation that the effect of lithium was not simple sedation but a true therapeutic effect on manic symptoms. Schou also recorded that some of the patients on long-term lithium treatment ‘did not feel quite their own self’, or, as one put it, felt ‘kept down’ by the medicine. Echoing that, some of today’s patients complain that lithium blunts their feelings, perceptions, and creativity. However, lithium was still difficult to administer, and blood levels were a matter of guesswork. The situation changed with the introduction of the Coleman flame photometer in 1958, which made the monitoring of plasma lithium levels much more precise than the previously used Beckman photometer.

As for lithium’s reception in Australia, by the mid-1950s, two more Australian doctors, in addition to Noack and Trautner, had published their experiences with lithium treatment. Bernard Glesinger, a psychiatrist at Claremont Hospital in Western Australia, gave lithium to 104 patients and found that ‘the calming effect on maniacal, excited, hyperactive and restless patients was most satisfying and appreciable’ [43] Another Australian doctor who had begun to use lithium, Max Margulies, of Lachlan Park Hospital in Tasmania, reported that several years earlier he had started treating ‘maniacal’ patients with lithium [44].

In a 1959 article that appeared in the journal Psychopharmacologia, Schou provided an update on lithium treatment with a paper under the title ‘Lithium in Psychiatric Therapy: Stock-Taking after Ten Years’ [45]. He reviewed the world’s psychiatric literature for accounts of manic patients treated with lithium and, as of early 1959, found 15 published reports, including Cade’s and his own. The total number of treated patients worldwide at that time was only 370. According to the authors of these reports, 304 of the patients (82%) had ‘improved’. Schou acknowledged that this rate of improvement needed to be viewed cautiously; the authors varied in how they defined both mania and its improvement and for how long they treated and observed the patients. Except for that in Schou’s study, the lithium treatment was ‘uncontrolled’, so it was not at all clear that lithium per se brought on the improvement.

**Figure 10:**
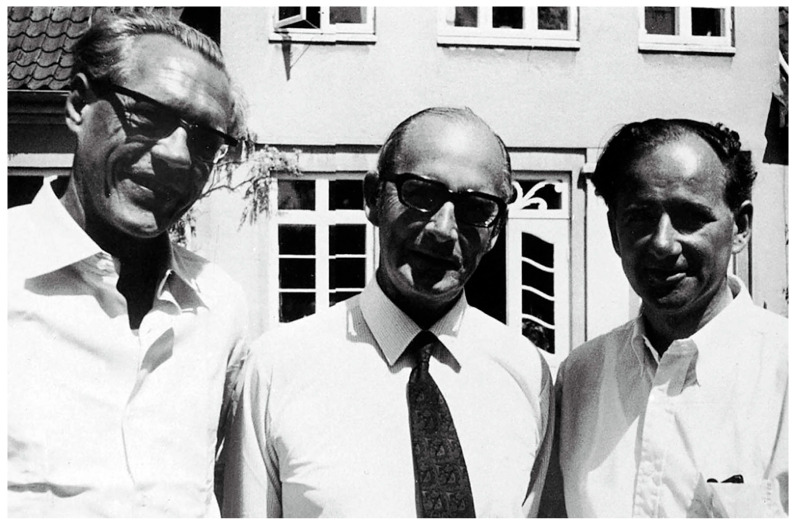
Left: Poul Christian Baastrup (1918–2002). Center: John Cade (1912–1980). Right: Mogens Schou (1918–2005). Copyright status: CC BY-SA 4.0.

The next year, Schou received letters from Geoffrey (Toby) Hartigan (1917–1968) from the UK and Poul Christian Baastrup (1918–2002; Figure 10) from Denmark. It is now known that for 25 years, Schou’s younger brother had suffered yearly episodes of depression that lasted several months each, rendered him unable to work, and, despite hospital stays and treatment with Electroconvulsive therapy (ECT) and medication, would recur every spring. Schou wondered if lithium was worth trying. Hartigan was encouraging, Schou followed his suggestion, and the results were almost miraculous. As the years went by, a number of family members, in addition to his brother, received lithium treatment with good results. Schou found it enormously gratifying that his research with lithium, in addition to improving the lives of millions and shedding light on the nature of manic-depressive illness, was also of direct benefit to his family.

A paper with Hartigan appeared in 1963 and was the first report to explicitly state that lithium could prevent severe depressive episodes [46,47]. Schou and Baastrup conducted a series of lithium experiments with ever stricter research rules. One year after Hartigan, in 1964, Baastrup published a report describing his first 11 patients and demonstrated the efficacy of lithium for the maintenance phase. In it, he mentioned Hartigan’s related observations [48]. Determined to find out if lithium really prevents episodes of mania and depression, in 1960, Baastrup embarked on a prospective study. The paper appeared in the February 1967 issue of the *Archives of General Psychiatry* (today named *JAMA Psychiatry*). In it, Baastrup and Schou showed that lithium had a striking prophylactic effect. Before lithium treatment, relapses had occurred on average every 8 months. Lithium treatment reduced the rate of relapse to only every 60–85 months. Before treatment with lithium, patients were spending, on average, 13 weeks per year in a manic state. Lithium treatment reduced this to less than 2 weeks per year. Almost 80% of the patients had no relapses at all during lithium treatment [49]. Eventually, they conducted a double-blind, placebo-controlled clinical trial with a proper design. It was published in 1970 in *The Lancet*, and it established lithium as an effective treatment for most people with Bipolar disorder. The story of that paper is interesting. Schou and his collaborators sent the paper to *The Lancet*, but its editor rejected it, feeling that ‘lithium had received enough publicity’. However, the intervention by W. Linford Rees, a prominent British psychiatrist, made the journal then agree to publish it, and it appeared in the 15 August 1970 issue [50].

In the meantime, in the US in 1960, Samuel Gershon received a Pfizer Fellowship that permitted him to join the Schizophrenia and Psycho-pharmacology Joint Research Project of the University of Michigan at the mental hospital in Ypsilanti, Michigan. While there, he carried his knowledge of lithium’s benefits to his Michigan associates and treated about 20 patients with lithium. He also gave lectures about lithium at the National Institute of Mental Health and elsewhere. The same year, along with Arthur Yuwiler (1927–2012) [51], also at Ypsilanti, they published the first North American paper on lithium [52]. In 1965, Gershon became director of the Neuropsychopharmacology Research Unit at New York University. There, he established a lithium clinic and research programs devoted to lithium and Bipolar disorder. Gershon and his collaborators did a series of studies that supported lithium’s specificity for the treatment of Bipolar disorder, identified some of lithium’s important side effects, including interference with the activity of the thyroid gland, and compared lithium with other psychiatric drugs [53,54,55].

In 1958, Ronald Fieve (1930–2018) was in the midst of his psychiatric training at New York’s Columbia University. He went on to carry out systematic studies of lithium as a treatment for both manic and depressive episodes. These studies confirmed lithium’s value in the treatment of mania and also verified its lack of effectiveness as a remedy for acute depression [56,57,58,59,60,61].

Throughout the 1970s, Fieve took it upon himself to increase the public’s awareness of manic-depressive illness and its successful treatment with lithium. He appeared on countless TV talk shows extolling the benefits of lithium, and in 1975, he published *Moodswing*, a book about manic depression for the lay public. It became a bestseller and sold over a million copies. In 1973, he invited Joshua Logan (1908–1988), the celebrated playwright, director, and producer (and also his patient), to appear with him on a televised American Medical Association (AMA) symposium about depression. Logan spoke candidly and in detail about his struggles with mania and depression and his recuperation with lithium. He went on to become a spokesperson for the value of lithium, and he and Fieve formed a persuasive duo on the talk show circuit. When, in 1973, they appeared with Barbara Walters on NBC’s Today, the station was flooded with phone calls for weeks afterward. Logan even wrote a book about his personal experience of living with mental illness [62].

**Figure 11:**
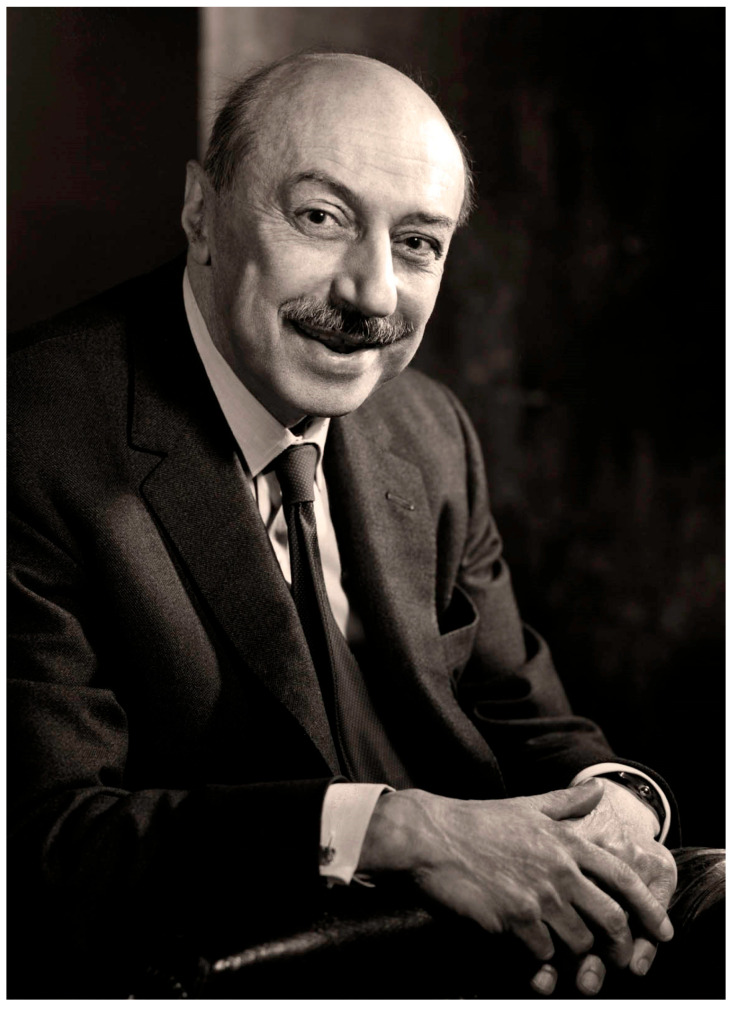
Aubrey Lewis (1900–1975), c. 1969, with permission from the National Portrait Gallery, London, https://npgimages.com/ (accessed on 7 April 2021).

For the next few years, there was significant academic opposition to the use of lithium as the standard treatment for Bipolar disorder (BD), and much emphasis was given to its toxicity. Two Maudsley psychiatrists, Aubrey Lewis (1900–1975; Figure 11) and Michael Shepherd (1923–1995), claimed that the idea, based on Schou’s studies, that lithium has a prophylactic effect was a ‘myth’.

They reproached Schou for not using a sufficiently rigorous control methodology. Some of the methodological fine points they raised were legitimate matters for debate and would be of particular relevance today. Aubrey Lewis was a professor of psychiatry and head of the Maudsley. He considered lithium treatment to be ‘dangerous nonsense’ while Michael Shepherd was also extremely negative towards it and suggested that lithium was toxic in mania and that claims of efficacy in the prevention of depression were based on ‘dubious scientific methodology’ [63,64,65,66,67,68]. The hard truth is that although later research confirmed the efficacy of lithium, at that time, in methodological terms, the Maudsley group was probably right. But the criticism took on an ad hominem tone. Critics also claimed that Schou was biased in favor of lithium’s preventive benefit because he had treated his brother with lithium, who had suffered from intractable recurrent depression, and was convinced that lithium had cured him, transforming his life. As Schou recalled, he has been called a naïve and biased ‘believer’ [32]. Rumors circulated in the Maudsley group that Schou himself had manic depression and was on lithium. His critics hinted that this explained his enthusiasm for the drug, his seemingly unreasonable bias in favor of it, and his conviction that it was of value in the absence of a proper, controlled clinical trial. When Schou heard about this sort of whispering, he unequivocally denied that he either had manic-depressive or was taking lithium (and there was no evidence whatsoever that he was) [69]. Still, the ad hominem arguments persisted, and they hurt.

However, it is interesting that in the frame of this debate in the 1970s, the exact opposite opinion was also advocated for; that is, there was no need for strict methodology and statistics, and the clinical impression was overwhelming and sufficient [70]. The ensuing storm, known among psychopharmacology insiders as the ‘Battle of Britain’, was resolved over the ensuing decade only after further studies by Schou and others confirmed the initial findings. Lithium treatment for Bipolar disorder was approved in 1961 in France, in 1966 in the UK, in 1967 in Germany, and in 1970 in Italy. A practical problem was that since there was nothing to patent and little profit to be expected, no drug firm clamored to produce it, and there seemed to be no one to apply for labeling to the US authorities. The problem seemed so impossible that the American College of Neuropsychopharmacology considered filing an application in its own name to the Food and Drug Agency (FDA) so that lithium could be legally prescribed. This eventually became unnecessary when, finally, three Pharma companies submitted applications to the FDA for approval of lithium. In April 1970, with guidance from the lithium task force, the FDA authorized lithium for the treatment of manic episodes. By then, 49 countries had already approved lithium. The US was the 50th. In 1974, the FDA extended its approval of lithium to include its use as a preventive agent for prophylactic treatment of manic-depressive illness [20]. Following the success of lithium treatment, valproate was introduced in 1966 [71] and later carbamazepine was introduced [72]. 

With its initial approval for use in Bipolar patients, research boomed, and data were accumulating. More studies established lithium and robustly linked it to the treatment of all phases of Bipolar disorder [20,41,49,50,73,74,75,76,77,78,79]. Later, Fred Goodwin (1936–2020) suggested it could also be useful in the treatment of depression as an add-on to antidepressants [80,81,82,83,84,85]. The recommended serum lithium levels were determined with certainty only in 1976 [86].

In a paper published in 1963, Schou proposed that lithium and some of the new antidepressants might be a novel class of psychiatric drugs, ones that not only relieved some of the symptoms of manic-depressive illness but treated all of its features, the disease itself. He named these drugs ‘mood-normalizers’ or ‘normothymotics’, from the Greek ‘θυμικό’ (‘thymiko’, meaning emotionality or feelings) and ‘θυμός’ (‘thymos’, meaning anger) [87]. The term ‘mood-normalizer’ was taken after the term ‘mood stabilizer’, which was used during the 1950s to refer to a combination of amphetamine plus a barbiturate to treat patients with neurotic instability but not patients with Bipolar disorder.

The acceptance of lithium was slow, and there were several reasons for this. The main issue was that since lithium is a natural substance, the Pharma industry was not interested in it because it could not be patented. As a result, there was no commercial interest, and no drug company promoted it. At the same time, the industry intensively marketed the psychiatric drugs that came on the scene during the 1950s, and the psychiatric community focused on these new agents. As for the place of lithium in the lay culture, it is interesting and important to mention its relationship with *Maude*, a popular TV sitcom, that devoted two episodes (on 26 January and 2 February 1976; ‘Maude’s Mood’, season 4, episodes 17 and 18) to manic-depressive illness. Norman Milton Lear (1922–2023), *Maude*’s producer, flew Nathan Kline to Los Angeles for a week to consult on the show. However, despite following Kline’s advice, the original plot included the psychiatrist explaining that this disorder is a ‘physical’ problem, a ‘chemical imbalance in the blood’, usually easy to control with lithium, and he proceeded to treat Maude with lithium successfully. In the episodes broadcast, lithium was never mentioned; instead, a vague reference to medication was made. At the end of the second episode, the psychiatrist Dr. Herbert Lester (portrayed by Tim O’Connor) says that Maude’s mood has been stabilized, so ‘now we can search for the real causes’, probably a necessary compromise to keep the prevailing psychoanalytical environment at least partially satisfied.

The advance publicity for the episodes mentioned Maude’s good response to lithium. Although the show’s reference to treatment was realistic and judicious, in 1976, lithium was still on shaky ground, and many psychiatrists, covering the spectrum from analysts to psychopharmacologists, objected to lithium’s positive portrayal. The ensuing controversy received a good deal of press coverage, with many professionals declaring that lithium was dangerous. In an article by Les Brown in the *New York Times*, Sam Gershon stated that ‘I have no serious reservations about lithium when used properly, but manic-depression is not a common disease, and that’s what it should be used for’. He and others worried that lithium’s favorable depiction on *Maude* could encourage a massive abuse of lithium and might encourage its use in all sorts of medical conditions (https://www.nytimes.com/1976/01/22/archives/lithium-use-in-maude-medical-issue.html) (accessed on 15 August 2025).

The most recent episode, however, took place in the 2010s. The hugely popular contemporary espionage thriller *Homeland*, which had its television debut in 2011 and finished its seventh season in 2018, features Carrie Mathison, a brilliant CIA agent played by Claire Danes, who has manic depression and handles her job and personal life well but only as long as she stays on lithium. In *Homeland*, lithium is not only clearly mentioned but it is also one of the key supporting players. Carrie confesses to a fellow agent: ‘It keeps me safe and sane’. The show’s depiction of her manic attacks, the crushing depressions that follow them, and lithium’s critical role in her life have been praised for their authenticity by both health professionals and similarly affected patients.

## 4. Discussion

Lithium was an unlikely hero in the history of psychiatric pharmacotherapy. The timing of its re-discovery, the way it was discovered, and its position in lay culture make its story almost extraordinary. The very idea that a single element that essentially does not exist in the human body could be therapeutic for a major medical disorder seems almost unbelievable. The fact that it appeared because it could facilitate the dissolving in the water of a pathological accumulation in the joints in vitro is also remarkable. Its history also points to the great importance, personal histories, sensitivities, and family problems of researchers, present throughout the history of medicine. This is a pattern that emerges again and again, like the case of the brother of Mogens Schou, and often explains how unbelievable leaps in logical thinking were made, which eventually were proven ‘lucky guesses’.

From a historical and sociopolitical standpoint, lithium constitutes one of the most valuable parts of psychiatry and one of its prides. It was the first material intervention proven efficacious in the treatment of mental disorders [88]. It received no funding or any kind of support from commercial interests and relied solely on the courage and persistence of researchers who worked against adverse conditions and even against the prevailing attitude of the time. Its use persists even today despite the lack of commercial interest and organized support.

## Data Availability

Not applicable.
